# Persistence of hepatitis B surface antibody until 7 years of age following administration of hexavalent and pentavalent vaccines in children at 2, 4, 6, and 18 months

**DOI:** 10.1016/j.jvacx.2024.100561

**Published:** 2024-09-20

**Authors:** Nasamon Wanlapakorn, Nasiri Sarawanangkoor, Donchida Srimuan, Thaksaporn Thatsanathorn, Sirapa Klinfueng, Yong Poovorawan

**Affiliations:** a Center of Excellence in Clinical Virology, Department of Pediatrics, Faculty of Medicine, Chulalongkorn University, Bangkok, Thailand; b The Academy of Science, The Royal Society of Thailand, Dusit, Bangkok 10300, Thailand

**Keywords:** Hepatitis, Pentavalent, Hexavalent, Vaccine, Antibody, Childhood

## Abstract

Thailand incorporated the hepatitis B (HepB) vaccine into the infant combination vaccine known as pentavalent wP-containing vaccines (DTwP-HB-Hib) and hexavalent aP-containing vaccines (DTaP-IPV-HB-Hib). We followed healthy children from the clinical trial (ClinicalTrials.gov NCT02408926) in which children were randomly assigned to receive either pentavalent or hexavalent vaccines for their primary series (administered at 2, 4, and 6 months) and first booster vaccination (at 18 months), following the monovalent HepB vaccine at birth. Blood samples were collected to evaluate the persistence of hepatitis B surface antibody (anti-HBs) at 3, 4, 5, 6 and 7 years of age. The results showed that at 7 years of age, a higher percentage of children in the hexavalent group maintained anti-HBs levels ≥ 10 mIU/mL compared to those in the pentavalent group (86.9 % vs. 59.7 %). This study showed good persistence of anti-HBs among hexavalent-vaccinated children 5.5 years after the last dose of the HepB vaccine.

## 1 Introduction

Combination vaccines for infants play a crucial role in promoting high levels of parental acceptance and vaccine coverage. This is attributed to the reduced number of individual injections required to achieve complete vaccination, while maintaining immunogenicity and safety. Thailand’s national policy initiated a pilot project to reduce hepatitis B virus (HBV) infection by incorporating the hepatitis B (HepB) vaccine into the Expanded Program on Immunization (EPI), beginning in 1988 in the two provinces of Chiang Mai and Chon Buri. The initial vaccination schedule was three doses administered at birth, 2 months, and 6 months of age. The second and third doses were administered concurrently with the diphtheria-tetanus-whole-cell pertussis (DTwP) vaccine, but at different injection sites. The recommended schedule was expanded to cover 12 provinces in 1990, and by 1992, it was integrated into the nationwide EPI [1]. The first change in the national policy of the HepB vaccine in Thailand occurred in 2005 when a combined DTwP-HB replaced the separate DTwP and HepB to reduce the number of individual injections, improve the efficiency of vaccine storage and delivery, and promote acceptance by parents, without compromising immunogenicity [2]. This change resulted in the change in the HepB vaccine schedule to four doses. The first dose was administered as a monovalent HepB vaccine at birth and the second to fourth doses were administered at 2, 4, and 6 months of age as a combined DTwP-HB [1]. The combined DTPw-HB vaccine was started in 12 provinces in 2005, increased to 24 provinces in 2006, and by 2008, the combined vaccine was implemented throughout the country.

The second change occurred in 2019 when *Haemophilus influenzae* type b (Hib) was integrated into the Thai EPI [3]. The combined pentavalent vaccine (DTwP-HB-Hib) in the Thai EPI (Shan-5, Sanofi Healthcare India Private Limited, Telangana, India) was administered to infants aged 2, 4, and 6 months, following the monovalent HepB vaccine at birth. In addition to DTwP-HB-Hib in the Thai EPI, the hexavalent DTaP-HB-Hib-IPV vaccine was also available as an optional vaccine (that is, the vaccine is not freely available, it must be purchased). Our previous study comparing anti-HBs levels induced by the pentavalent DTwP-HB-Hib vaccine (Quinvaxem, Crucell-Janssen, Incheon, South Korea) and the hexavalent DTaP-HB-Hib-IPV (Infanrix hexa, GSK, Rixensart, Belgium) showed that higher percentages of infants achieving hepatitis B surface antibody (anti-HBs) levels ≥ 10 mIU/mL were found in the hexavalent group than the pentavalent group at 2 years of age [4]. However, data comparing the longitudinal kinetics of anti-HBs between pentavalent and hexavalent-vaccinated cohorts was lacking, so we extended the follow-up of children from the previous trial until children reached the age of seven [4]. Data on anti-HBs levels from birth to two years of age were previously published [4]. The primary objective of the current study is to assess the persistence of anti-HBs up to the age of seven, which corresponds to 5.5 years post-administration of the last HepB vaccination dose, in children who previously received pentavalent Quinvaxem or hexavalent Infanrix hexa vaccine administered at 2, 4, 6 and 18 months of age, after monovalent HepB vaccine at birth.

## 2 Materials and Methods

### 2.1 Study design and study vaccine

This study was a long-term follow-up of children who participated in a previous trial (ClinicalTrials.gov NCT02408926) [4]. In brief, 370 pregnant women who attended the antenatal care clinic at King Chulalongkorn Memorial Hospital, Bangkok, Thailand, and consented to Tdap vaccination (Boostrix, GSK, Rixensart, Belgium) during the third trimester were included in the study. Healthy full-term and late preterm infants were randomly assigned to receive either the hexavalent aP-containing vaccine (Infanrix hexa; hexavalent group) or the pentavalent wP-containing vaccine (Quinvaxem; pentavalent group). During the same period, a comparison group of 79 infants, born to mothers vaccinated with TT or dT but not Tdap, were recruited after birth from the same hospital as the hexavalent and pentavalent groups. This group received the pentavalent vaccine (Quinvaxem) for primary and first booster vaccination (hereafter referred to as the EPI pentavalent group). The EPI pentavalent group represented the immune response of children under the national vaccination policy when Tdap vaccination during pregnancy was not incorporated, and pentavalent wP-containing vaccines were used for primary and booster immunization.

The components of the hexavalent and pentavalent vaccines were previously reported [4], [5]. The amount of hepatitis B surface antigen (HBsAg) in both the hexavalent and pentavalent vaccines was equal (10 µg of HBsAg). All children received an intramuscular monovalent HepB vaccine at birth and pentavalent or hexavalent vaccines at 2, 4, 6 months of age (primary vaccination) and at 18 months of age (first booster vaccination). Infants born to HBsAg-positive mothers also received a HepB immunoglobulin (HBIG) at birth and a monovalent HepB vaccine at one month of age. In the previous study, the children were followed up at birth, month 7, month 18, month 19, and month 24 (2 years of age) [4].

All eligible children who completed their month-24 follow-up visits were contacted for participation in the present study. In this study, children were scheduled for blood sample visits at 3, 4, 5, 6 and 7 years of age. This long-term follow-up study was conducted between May 2018 and November 2023 at the Clinical Research Unit, Center of Excellence in Clinical Virology, Department of Pediatrics, Faculty of Medicine, Chulalongkorn University, Bangkok, Thailand. This study was approved by the Institutional Review Board of the Faculty of Medicine of Chulalongkorn University (IRB no. 084/60 and 173/63) and was registered with the Thai Clinical Trial Registry (TCTR20231017001). Written informed consent was obtained from parents or legal guardians, and written assent was obtained from children at the age of 7 before enrollment.

### 2.2 Laboratory tests

Venous blood samples (6 mL) from all participants were collected at the ages of 3, 4, 5, 6 and 7 years. Anti-HBs were evaluated by Chemiluminescent Microparticle Immunoassay (CMIA) using an automated ARCHITECT system (Abbott Laboratory, Wiesbaden, Germany) as previously described [4]. The cut-off value for anti-HBs seroprotection was ≥ 10 mIU/mL.

### 2.3 Statistical analysis

Anti-HBs levels are presented as geometric mean concentration (GMC) with a 95 % confidence interval (CI). The proportions of seroprotected individuals were displayed as number and percentages. The conventional *t*-test was used to compare the GMC of anti-HBs between the different study groups. This analysis was carried out using SPSS statistic software. The p-value < 0.05 indicates statistical significance.

## 3 Results

### 3.1 Demographic characteristics of the study participants

At 24 months of age, 295 participants were eligible and contacted for participation in this long-term follow-up study (125 from the hexavalent group, 113 from the pentavalent group, and 57 from EPI pentavalent group). A total of 119 participants in the hexavalent group, 111 participants in the pentavalent group, and 57 participants in the EPI pentavalent group were included (Table 1). A total of 92 participants in the hexavalent group, 77 participants in the pentavalent group, and 43 participants in the EPI pentavalent group completed this study at 7 years of age. Males and females were equally distributed in the hexavalent (48.8 % male and 51.2 % female) and pentavalent groups (49.6 % male and 50.4 % female), while in the EPI pentavalent group, there were slightly more male than female participants (61.4 % male versus 38.6 % female). The baseline characteristics of the children was previously published in the original study that aims to evaluate the long-term immunogenicity of pertussis-containing vaccines in this cohort [5]. There were no statistically significant differences in mean weight and height between the groups at any of the time points studied.

**Table 1:** Comparison of geometric mean concentration (GMC) and seroprotective level (anti-HBs ≥ 10 mIU/mL) from birth to 7 years (month 84) in hexavalent, pentavalent and EPI pentavalent groups.

**Age (in months)**	**Hexavalent**			**Pentavalent**			**EPI Pentavalent**		
	**N**	**GMC (mIU/mL) (95 % CI)**	**Protective level n (%)**	**N**	**GMC (mIU/mL) (95 % CI)**	**Protective level n (%)**	**N**	**GMC (mIU/mL) (95 % CI)**	**Protective level n (%)**
**Birth**	137	45.1 (22.5–90.4)	33 (24.1)	127	102.9 (49.6–213.7)	40 (31.5)	N/A	N/A	N/A
month 7	131	2962 (2334–3759)	130 (99.2)	127	308 (249.2–380.4)	126 (99.2)	65	236 (167.4–332.1)	64 (98.5)
month 18	134	200 (155.3–258.2)	130 (97)	130	29 (23.3–36.1)	104 (80)	47	19 (11.7–31)	31 (66)
month 19	133	5906 (4084–8542)	133 (100)	126	521 (380–715)	125 (99.2)	64	377 (227.2–624.2)	60 (93.8)
month 24	121	1790 (1232–2603)	119 (98.3)	111	89.3 (63–127)	96 (86.5)	56	86.8 (47.9–157.2)	43 (76.8)
month 36	119	564.3* (385.6–826.0)	114 (95.8)	111	36.9 (25.5–53.3)	79 (71.2)	57	31.5 (17.9–55.2)	39 (68.4)
month 48	121	320.3* (221.6–462.8)	115 (95)	109	28.2 (19.6–40.6)	76 (69.7)	55	18.7 (10.9–32.1)	34 (61.8)
month 60	104	243.5* (165.4–358.4)	98 (92.5)	94	18.0 (12.1–26.7)	58 (61.7)	48	12.8 (7.4–22.2)	26 (54.2)
month 72	101	162.5* (112.4–234.8)	91 (90.1)	87	15.8 (10.7–23.1)	53 (60.9)	46	11.0 (6.5–18.7)	22 (47.8)
Month 84	92	143.2* (93.4–219.6)	80 (86.9)	77	14.8 (9.6 – 22.7)	46 (59.7)	43	9.6 (5.3–17.3)	21 (48.8)

GMC; geometric mean concentration, CI; confidence interval, EPI; Expanded Program on Immunization

*indicates p < 0.001 in the hexavalent groups compared to the pentavalent and EPI pentavalent groups.

### 3.2 Persistence of anti-HBs and seroprotective levels in children between 3 and 7 years of age

**Fig. 1:**
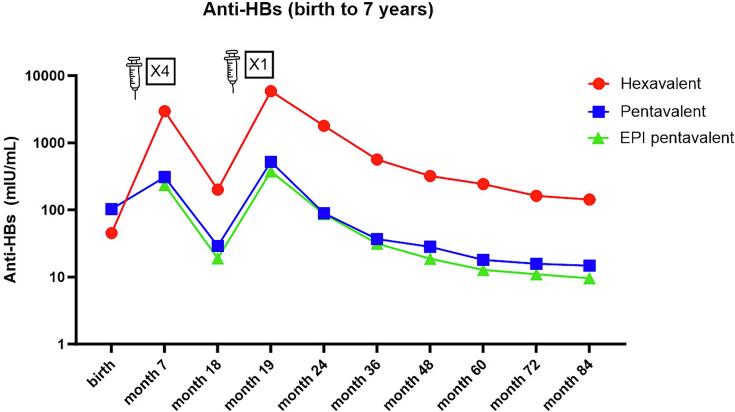
Geometric mean concentrations (GMCs) of anti-HBs from birth to 7 years among the hexavalent, pentavalent, and EPI pentavalent groups. Data from birth to month 24 were previously published [4]. Syringes indicate the time point of primary and booster vaccinations. The number of doses received was indicated in the box next to the syringes.

As shown in Table 1, following primary and first booster vaccinations, the hexavalent-vaccinated children possessed significantly higher anti-HBs levels than the pentavalent and EPI pentavalent groups at months 19 (one month after booster, p-value < 0.001). Although anti-HBs levels waned over time, the hexavalent group demonstrated higher anti-HBs GMCs (approximately by one log scale) at each time point after booster, compared to the pentavalent and EPI pentavalent groups (Fig. 1). No notable differences were observed in the anti-HBs GMCs between the pentavalent and EPI pentavalent groups at any of the tested time points.

In terms of seroprotection rate, anti-HBs levels ≥ 10 mIU/mL were achieved in 100 % of the children in the hexavalent group, 99.2 % in the pentavalent group and 93.8 % of those in the EPI pentavalent group one month after the booster. The percentages of children with seroprotective concentration decreased over time, as illustrated in Table 1. Nevertheless, a significantly higher proportion of children in the hexavalent group maintained anti-HBs levels ≥ 10 mIU/mL compared to the pentavalent and EPI pentavalent groups at 3, 4, 5, 6, and 7 years of age.

## 4 Discussion

This study was a long-term follow-up study that examined the durability of anti-HBs induced by hexavalent or pentavalent vaccines administered to children at 2, 4, 6, and 18 months of age, following administration of a monovalent HepB vaccine at birth. Participants in our study group received a cumulative of 5 doses of the HepB-containing vaccine. In our study, both the proportion of children with seroprotective anti-HBs concentrations and their GMCs were significantly lower among those immunized with the pentavalent Quinvaxem vaccine than those immunized with the hexavalent Infanrix hexa vaccine, despite the fact that there was the same amount of HBsAg in both vaccines. A previous study showed that a lower amount of HBsAg (i.e. 5 µg versus10 µg of HBsAg) resulted in lower immunogenicity [6]. In theory, an equivalent amount of antigen should elicit comparable immune responses. However, we postulated that practical variations in real-world settings, such as differences in antigen quality, adjuvant type, route of administration, and vaccine formulation and manufacturing process, could potentially affect its ability to stimulate an immune response.

A previous study in children primed in infancy and boosted in the second year of life with DTaP-HB-IPV-Hib showed that at 7–9 years of age, 72.2 %–78.0 % of subjects continued to have anti-HBs concentrations ≥ 10 mIU/mL [7], [8]. Likewise, 86.9 % of the hexavalent-vaccinated children in our study maintained seroprotective concentrations at 7 years of age. The higher percentage of children with seroprotective anti-HBs concentrations in our hexavalent group could be due to the higher number of total HepB vaccine doses (5 doses in the present study versus 4 doses in others). Data on long-term persistence of anti-HBs after DTwP-HB-Hib are limited. However, our group conducted a study on long-term persistence of anti-HBs after DTwP-HB which found that when children received the DTwP-HB vaccine at 2, 4, 6, and 18 months of age after a monovalent HepB vaccine at birth, the seroprotective concentration of anti-HBs was maintained in 90.9 % of subjects at 7 years of age [9]. This is higher than the pentavalent and EPI pentavalent group in this study (only 59.7 % and 48.8 %, respectively), but similar to the proportions achieved in the hexavalent group (86.9 %).

Currently, the combined vaccine in the Thai EPI is DTwP-HB-Hib (Shan-5). It is essential to note that the geometric mean concentrations of anti-HBs induced by EPI Shan-5 and Infanrix hexa were comparable but higher than those induced by Quinvaxem one month after primary immunization [3]. Furthermore, our previous study showed that, at 18 months of age, a greater percentage of children who received EPI Shan-5 and Infanrix hexa maintained anti-HBs levels ≥ 10 mIU/mL compared to those who received Quinvaxem [3].

Previous data have shown that, at 5–13 years after the third dose of HepB immunization, approximately 41 %-73.5 % of vaccinated subjects maintained seroprotective anti-HBs concentration, with a GMT between 2.99–64 mIU/mL [10], [11], [12]. Although anti-HBs waned over time, previous studies found that strong immunological memory persists for more than 10 years after three-dose primary HepB immunization during childhood [10], [11], [12]. Thus, booster doses do not seem necessary to ensure long-term protection.

This study has some limitations. First, it did not evaluate immune memory to hepatitis B in children of all groups. Secondly, the small sample size within the EPI pentavalent group raises caution about the interpretation of statistical analyses. Besides, the EPI pentavalent cohort is entirely distinct and not drawn from the same initial pool which underwent randomization. Lastly, the dropout rate from the previous trial might introduce some unknown biases. Additional follow-up of children, particularly those who received pentavalent vaccines, is required to determine whether sufficient immunological memory persists or whether a booster might be needed later in life to maintain lifelong protection.

In conclusion, this study demonstrated a greater persistence of anti-HBs among children vaccinated with the hexavalent vaccine compared to those who received the pentavalent vaccine, as observed at 5.5 years after the last dose of the HepB vaccine. Ongoing research is being conducted to evaluate immunological memory of hepatitis B and determine whether a booster is needed.

Data will be available upon request.Funding

This work was funded by the Research Grant for Talented Young Researchers (N41A640104) of the National Research Council of Thailand (NRCT), the Health Systems Research Institute (HSRI), the Ratchadaphisek Somphot Fund, Faculty of Medicine, Chulalongkorn University and the Research Grant from the Center of Excellence in Clinical Virology, Faculty of Medicine, Chulalongkorn University.

## CRediT authorship contribution statement

**Nasamon Wanlapakorn:** Writing – review & editing, Writing – original draft, Supervision, Funding acquisition, Formal analysis, Data curation, Conceptualization. **Nasiri Sarawanangkoor:** Writing – original draft, Formal analysis, Data curation. **Donchida Srimuan:** Project administration. **Thaksaporn Thatsanathorn:** Project administration. **Sirapa Klinfueng:** Methodology, Investigation. **Yong Poovorawan:** Writing – review & editing, Supervision, Funding acquisition, Formal analysis, Data curation, Conceptualization.

## Declaration of competing interest

The authors declare that they have no known competing financial interests or personal relationships that could have appeared to influence the work reported in this paper.

## Data Availability

Data will be made available on request.
